# A Cambrian–Ordovician Terrestrialization of Arachnids

**DOI:** 10.3389/fgene.2020.00182

**Published:** 2020-03-11

**Authors:** Jesus Lozano-Fernandez, Alastair R. Tanner, Mark N. Puttick, Jakob Vinther, Gregory D. Edgecombe, Davide Pisani

**Affiliations:** ^1^School of Biological Sciences, University of Bristol, Bristol, United Kingdom; ^2^School of Earth Sciences, University of Bristol, Bristol, United Kingdom; ^3^Department of Biology and Biochemistry, Milner Centre for Evolution, University of Bath, Bath, United Kingdom; ^4^Department of Earth Sciences, Natural History Museum, London, United Kingdom

**Keywords:** Arachnida, Chelicerata, terrestrialization, Cambrian, molecular clocks, diversification

## Abstract

Understanding the temporal context of terrestrialization in chelicerates depends on whether terrestrial groups, the traditional Arachnida, have a single origin and whether or not horseshoe crabs are primitively or secondarily marine. Molecular dating on a phylogenomic tree that recovers arachnid monophyly, constrained by 27 rigorously vetted fossil calibrations, estimates that Arachnida originated during the Cambrian or Ordovician. After the common ancestor colonized the land, the main lineages appear to have rapidly radiated in the Cambrian–Ordovician boundary interval, coinciding with high rates of molecular evolution. The highest rates of arachnid diversification are detected between the Permian and Early Cretaceous. A pattern of ancient divergence estimates for terrestrial arthropod groups in the Cambrian while the oldest fossils are Silurian (seen in both myriapods and arachnids) is mirrored in the molecular and fossil records of land plants. We suggest the discrepancy between molecular and fossil evidence for terrestrialization is likely driven by the extreme sparseness of terrestrial sediments in the rock record before the late Silurian.

## Introduction

Arachnids are an important group of terrestrial arthropods, including the familiar ticks, mites, spiders, and scorpions, together with pseudoscorpions, camel spiders, vinegaroons, whip spiders, and a few other groups. Arachnids are important predatory arthropods across almost every conceivable terrestrial habitat. While ticks are ectoparasites that affect humans and livestock, spiders are ecologically the most successful arachnids and as predators consume vast quantities of insects. Thus, understanding when arachnids colonized land and diversified is of interest from a macroevolutionary and macroecological perspective.

Arachnids are chelicerates, together with the marine horseshoe crabs (Xiphosura) and sea spiders (Pycnogonida). They are the most speciose clade in Chelicerata, with more than 112,000 described extant species. Together with hexapods and myriapods, arachnids represent one of three distinct and ancient events of arthropod terrestrialization (terrestrial isopods are a younger addition to the continental arthropod biota). While arachnids have traditionally been considered monophyletic and terrestrial (apart from secondarily marine mites: Pepato et al., 2018) this picture has been challenged at different times. Scorpions were long thought to be the sister group of all other extant arachnids (Weygoldt and Paulus, 1979) or most closely allied to the aquatic “sea scorpions,” the eurypterids (Dunlop and Webster, 1999). Early fossil scorpions have been interpreted as aquatic, and in some cases even as marine. These phylogenetic hypotheses and interpretations of fossil ecology have been seen as requiring independent events of terrestrialization. Some of these views have been overturned by strong molecular (Regier et al., 2010; Sharma et al., 2014; Sharma and Wheeler, 2014; Leite et al., 2018; Ballesteros and Sharma, 2019; Lozano-Fernandez et al., 2019) and morphological (Garwood and Dunlop, 2014; Klußmann-Fricke and Wirkner, 2016) evidence for scorpions being nested within the Arachnida as the sister group of the other arachnids with book lungs, the Tetrapulmonata. Indeed, detailed correspondences in book lung morphology between scorpions and tetrapulmonates support their homology (Scholtz and Kamenz, 2006). The supposed aquatic mode of life of various fossil scorpions has also been questioned on both morphological and geological grounds (Dunlop et al., 2008; Kühl et al., 2012). Another challenge to a single terrestrialization event in arachnids came from analyses of phylogenomic datasets, which have often recovered the marine Xiphosura to be nested *within* Arachnida (Ballesteros and Sharma, 2019). This remains a contentious issue, as other phylogenomic analyses have yielded trees in which Arachnida is monophyletic (Lozano-Fernandez et al., 2019).

Most chelicerate lineages are predatory components of a diverse range of ecosystems, and the rock record attests to their presence in both earlier Paleozoic marine settings (Legg, 2014) and through into the Mesozoic and Cenozoic, which witnessed a prolific diversification of spiders and other terrestrial arachnids (Penney, 2003; Selden et al., 2009). The terrestrial rock record prior to the Silurian is very sparse (Kenrick et al., 2012) and has presented some apparent discordances when investigating myriapod (Fernández et al., 2018), hexapod (Lozano-Fernandez et al., 2016), and plant divergence times. While the body fossil record of terrestrial plants and arthropods does not extend much further back than the Silurian (∼443–419 Ma), molecular clock estimates go back to the Ordovician (485–443 Ma) and Cambrian (538–485 Ma) (Morris et al., 2018b; Lozano-Fernandez et al., 2016). However, likely plant spores with desiccation-resistant adaptations extend back to the middle Cambrian (Gensel, 2008). The fossil record presents no unequivocal evidence for crown-group arachnids before the Silurian. The oldest crown-group arachnids are of Silurian age (stem-group Scorpiones in the Llandovery), followed by the extinct Trigonotarbida in the late Silurian (early Přídolí), Acariformes and Opiliones in the Early Devonian (Pragian), and Pseudoscorpiones in the Middle Devonian (Givetian). Several other arachnid orders first appear in the Carboniferous, including Araneae, Uropygi, Amblypygi, and Ricinulei. In contrast to a picture of scattered branches of the arachnid crown-group first appearing in the Siluro–Devonian, older representatives of the arachnid lineage are stem-group Arachnida, and are marine shoreline or brackish water/estuarine forms rather than being terrestrial. These include Chasmataspida and Eurypterida, the earliest members of which date to the Miaolingian Series of the Cambrian (Drumian Stage) and the Late Ordovician (Sandbian), respectively (Dunlop et al., 2003; Dunlop, 2010; Wolfe et al., 2016). Xiphosura-like chelicerates have a good fossil record, showing considerable morphological stasis, with marine stem-group representatives of Xiphosura such as *Lunataspis* being documented from the Late Ordovician, ca. 445 Ma (Rudkin et al., 2008), and a species from the Early Ordovician (Tremadocian) of Morocco (Van Roy et al., 2010) extends the lineage’s history even deeper. With such a deep history revealed by the fossil record, any inferred phylogenetic position for Xiphosura within terrestrial arachnids would imply that the marine ecology of this lineage should be a secondary acquisition. Although such a scenario is paleontologically unlikely, molecular studies have often recovered horseshoe crabs in highly derived clades of arachnids, such as sister groups to Opiliones or Palpigradi (Pepato and Klimov, 2015), Ricinulei (Sharma et al., 2014; Ballesteros and Sharma, 2019), or Scorpiones and Araneae (von Reumont et al., 2012; Roeding et al., 2009; Sanders and Lee, 2010).

As with other terrestrial groups, molecular dating has recovered old dates for the origin and main diversification of arachnids. As part of wider campaigns investigating arthropods using just a few arachnid representatives, Rota-Stabelli et al. (2013), Wheat and Wahlberg (2013), and Lozano-Fernandez et al. (2016) recovered dates for the origin of Arachnida with credibility intervals bracketed between the Cambrian, in the first two studies, and Ordovician in the latter. Recently, Ballesteros and Sharma (2019) reported a chelicerate molecular phylogeny in which when they constrained Arachnida to be monophyletic inferred an Ediacaran origin for the group. Consequently, there are significant geochronological discrepancies, particularly for terrestrial lineages, between the molecular clock-based studies and the younger dates suggested by the first appearances of fossils. As fossils do not inform on the age of origin of clades (Signor and Lipps, 1982), but rather provide minimum ages of divergence (Donoghue and Benton, 2007), clock-based methods are required to approach an accurate evolutionary timescale.

Focusing on the favored topology of Lozano-Fernandez et al. (2019) that recovers both Arachnida and Acari as monophyletic groups, we here estimate the divergence time of arachnids. To calibrate the molecular clock, we use a carefully selected and expanded set of 27 fossil constraints across the tree.

## Materials and Methods

### Phylogenetic Reconstruction

The molecular supermatrix used here is composed of 89 species, 75 of them being chelicerates, with 14 other panarthropod species as outgroups. This matrix (Lozano-Fernandez et al., 2019, matrix A after exclusion of six unstable taxa) is a concatenation of 233 highly conserved and slow-evolving genes retrieved from transcriptomic data (45,939 amino acid positions and 78,1% complete). To evaluate the robustness of the results to an alternative topology, we also performed a divergence-date analysis in which Arachnida was non-monophyletic, with Xiphosura nested inside the arachnids (Lozano-Fernandez et al., 2019, matrix B containing 95 taxa). The phylogenetic trees were inferred using PhyloBayes MPI v.4.1 (Lartillot et al., 2013) under the site-heterogeneous CAT–GTR + Γ model of amino acid substitution (Lartillot and Philippe, 2004). Convergence was assessed by running two independent Markov chains and using the bpcomp and tracecomp tools from PhyloBayes to monitor the maximum discrepancy in clade support (maxdiff), the effective sample size (effsize), and the relative difference in posterior mean estimates (rel_diff) for several key parameters and summary statistics of the model. We ran the analysis for 10,000 cycles and discarded as “burn-in” the first 3,000 generations.

### Molecular Clock Analyses

Divergence time estimation was performed using PhyloBayes 3.3f (serial version) (Lartillot et al., 2009). We fixed the topology following Lozano-Fernandez et al. (2019), see previous section. We compared the fit of alternative, autocorrelated (CIR model – Lepage et al., 2006, 2007) and uncorrelated (uncorrelated gamma multipliers model; UGAM – Drummond et al., 2006), relaxed molecular clock models generating ten different random splits replicates and performing cross-validation analyses (see PhyloBayes manual for details). The tree was rooted on the Onychophora–Euarthropoda split. A set of 27 fossil calibrations and 1 node constrained by a maximum age (see Table 1 and Supplementary Data Sheet S4 for justifications) was used. We imposed a soft maximum of 559 Ma for the onychophoran–euarthropod split based on trace fossils in the White Sea/South Australian Ediacaran. This uses the radiometric date of 558+/− 1 Ma from Martin et al. (2000) for strata at which body fossils such as *Kimberella*, a putative total-group bilaterian metazoan (Martin et al., 2000; Benton et al., 2015), occur. Metazoan trace fossils in the White Sea/South Australian Ediacaran indicate suitable preservation for arthropod traces, were they present. We regard this to be a conservative soft maximum, as there is no body or trace fossil evidence for arthropods in the Ediacaran. A minimum for the divergence of onychophorans and arthropods is set by the earliest *Rusophycus* traces (total-group Arthropoda), dated to a minimum of 528.8 Ma following Wolfe et al. (2016). To allow the analysis to explore younger ages and prevent having posterior ages being much older than the fossil record, we also set maximum constraints on a few of the deepest calibrations within Euarthropoda (Ho and Phillips, 2009; Wolfe et al., 2016; Morris et al., 2018a). We infer that crown-group Mandibulata and Chelicerata do not predate the oldest fossil evidence for arthropods (*Rusophycus*; see above) and set soft maxima for each of this pair of sister taxa at the base of the Cambrian (538.8 Ma following Linnemann et al. (2019). Within Arachnida, we infer Acari and Arachnopulmonata do not predate the oldest body fossils of crown-group Chelicerata, using *Wisangocaris barbarahardyae* and its date of 509 Ma as a soft maximum following Wolfe et al. (2016). The amino acid substitution model used to estimate branch lengths was the CAT–GTR + Γ model, as in the phylogenetic analyses of Lozano-Fernandez et al. (2019). All analyses were conducted using soft bounds with 5% of the probability mass outside the calibration interval. A birth–death model was used to define prior node ages. Analyses were run under the priors to evaluate the effective joint priors induced by our choice of calibrations and root maxima. Convergence was considered achieved with *tracecomp* statistics dropping below 1 for all relative difference scores, and all effective sample sizes being above 50, for all chain parameters. The time-scaled phylogenies were plotted using the package MCMCtreeR, which allows the display of full posterior distributions on nodes and the inclusion of the geological timescale (Puttick, 2019). We included as supplementary data the chronograms, the guiding trees and the calibration file used in PhyloBayes, the subset of sampled timescaled trees used to generate the posterior distributions shown on the figures (“datedist” PhyloBayes file), and the two molecular matrices (Supplementary Data Sheet S5).

**TABLE 1:** Set of numbered fossil calibrations with minima and maxima.

Number	Clade constrained	Calibration	Maxima	Minima
1	Chelicerata	*Wisangocaris barbarahardyae*	538.8	509
2	Euchelicerata	*Chasmataspis*-like resting traces		500.5
3	Arachnopulmonata	*Palaeophonus loudonensis*	509	432.6
4	Opiliones	*Eophalangium sheari*	509	405
5	Acari	*Protocarus crani*	509	405
6	Sarcoptiformes	*Protochthonius gilboa*		382.7
7	Pedipalpi	*Parageralinura naufraga*		319.9
8	Palpatores	*Macrogyrion cronus*		298.75
9	Araneae	*Palaeothele montceauensis*		298.75
10	Avicularoidea	*Rosamygale grauvogeli*		240.5
11	Xiphosurida	*Tachypleus gadeai*		236
12	Synspermiata	*Eoplectreurys gertschi*		158.1
13	Entelegynae	*Mongolarachne jurassica*		158.1
14	Araneoidea	Unnamed Linyphiinae		129.41
15	Bipectina	*Cretamygale chasei*		125
16	Bothriuoidea + Scorpionoidea + “Chactoidea”	*Protoischnurus axelrodurum*		112.6
17	Cyphopthalmi	*Palaeosiro burmanicum*		98.17
18	Laniatores	*Petrobunoides sharmai*		98.17
19	Metastriata	*Amblyomma birmitum*		98.17
20	Buthida	*Uintascorpio halandrasorum*		48.5
21	Lobopodia	*Rusophycus avalonensis*	559	528.82
22	Altocrustacea	*Yicaris dianensis*		514
23	Progoneata	*Casiogrammus ichthyeros*		426.9
24	Chilopoda	*Crussolum* sp.		416
25	Hexapoda	*Rhyniella praecursor*		405
26	Aparaglossata	*Westphalomerope maryvonnae*		313.7
27	Onychophora	*Cretoperipatus burmiticus*		100
	Mandibulata		538.8	

### Rate of Molecular Evolution and Diversification

We estimated rates of molecular evolution within Chelicerata using two different methods. For the first method, we modeled the rates of molecular evolution on a fixed tree topology constrained to the timetree relationships. On this tree we estimated relative branch lengths under the C60 model + Γ in IQTree (Nguyen et al., 2015). We then divided these relative branch lengths by the timetree lengths to provide an estimate of absolute molecular rates through time. For the second method, we inferred ancestral estimates of the amino acid sequence on the fixed timetree, again using the C60 model + Γ in IQTree. We divided the sum of gross amino acid changes between ancestral and descendant nodes by absolute time to obtain per-branch rates of change.

We estimated speciation and extinction rates on the fixed timetree by using a Bayesian episodic diversification rate model in RevBayes 1.0.10 (Höhna et al., 2016). This model estimates piece-wise rates of speciation and extinction on a phylogeny through time (Stadler, 2011; Höhna, 2015). Within each bin, rates of speciation are equal but can differ between bins. The initial episodic speciation and extinction rate was sampled from a log-uniform distribution *U*(−10,10). Moving backward in time for each distinct time bin, the model samples speciation and extinction rate from a normal distribution with the mean inherited from the value of the previous bin so rates are auto-correlated. Each normal distribution has a standard deviation inferred from an exponential hyper-prior of mean 1. In this manner, the model follows a Brownian motion pattern of rate change through time. To incorporate incomplete sampling in the model, we provided estimates of the known extant species numbers to complement the diversity shown in the tree using empirical taxon sampling by providing estimate diversity represented by each tip on our incomplete time tree. This empirical taxon-sampling approach is believed to produce less biased estimates of speciation and extinction parameters compared to diversified taxon sampling (Höhna, 2014). We used the values of the described extant species from Zhang (2013): Pycnogonida (1346); Xiphosura (4); Ricinulei (77); Opiliones (6571); Solifugae (1116); Acariformes (42233); Parasitiformes (12385); Pseudoscorpiones (3574); Scorpiones (2109); Uropygi (119); Amblypygi (172); and Araneae (44863). As we tested for the presence of early high rates compared to later times rather than differences in geological time units (e.g., Period), we assumed there were 10 equally sized time intervals which can potentially possess distinct speciation and extinction rates. We included as supplementary data the input files used for the diversification analyses (Supplementary Data Sheet S5).

## Results

### Molecular Divergence Time Estimation

**FIGURE 1:**
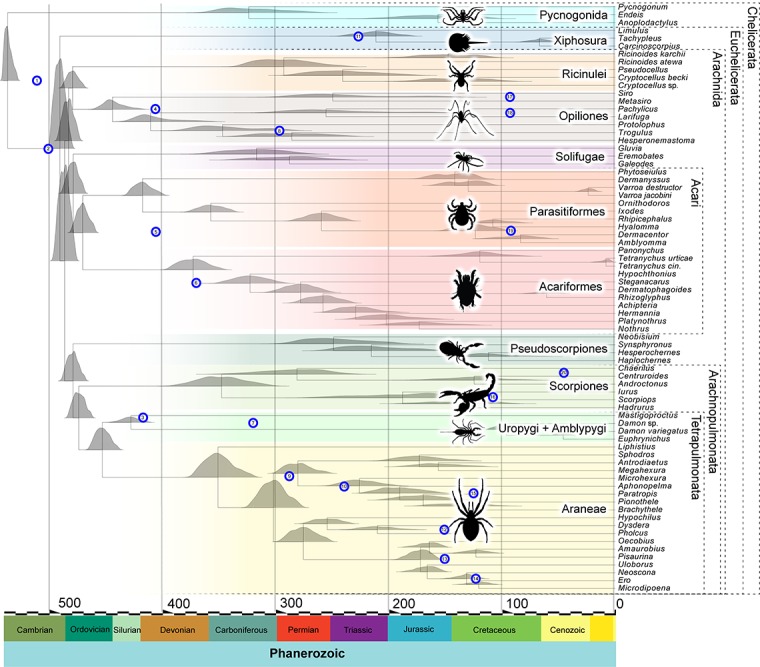
Chelicerate divergence times in the molecular clock analysis (outgroups not shown). Divergence times shown are obtained under the CIR autocorrelated, relaxed molecular clock model. Nodes in the tree represent average divergence times. The density plots represent the posterior distributions from the considered node. The numbered blue circles represent the age of the fossil calibrations and are located at a height corresponding to the node they are calibrating (see Table 1). In the timescale on the *X* axis, numbers represent millions of years before the present.

The topology used for the molecular clock analyses here is obtained from Lozano-Fernandez et al. (2019). In this tree Chelicerata and Euchelicerata are monophyletic, with the horseshoe crabs retrieved as the sister group of monophyletic Arachnida. Bayesian cross-validation indicates that the autocorrelated CIR model (Lepage et al., 2006, 2007) most optimally fits the data – cross-validation score = 32.00 ± 9.44 – against UGAM (with all ten replicates supporting CIR as best). Accordingly, divergence time estimation was performed using the Autocorrelated CIR model and results within Chelicerata are presented in Figure 1, the full chronogram in Supplementary Data Sheet S1 and the retrieved ages in Table 2.

**TABLE 2:** Molecular divergence times for arthropod crown-group lineages.

Taxon	Mean age (Ma)	95% Credibility interval	Taxon	Mean age (Ma)	95% Credibility interval
Lobopodia	553	559–544	Pycnogonida	316	359–261
Euarthropoda	546	551–536	Xiphosura	210	239–179
Mandibulata	535	539–526	Ricinulei	294	338–236
Myriapoda	516	524–505	Opiliones	440	457–412
Chilopoda	429	460–389	Solifugae	318	361–258
Diplopoda	506	518–489	Acari	469	481–475
Pancrustacea	486	501–471	Parasitiformes	417	434–399
Hexapoda	422	448–400	Acariformes	374	396– 347
Chelicerata	535	540–527	Pseudoscorpiones	244	290–172
Euchelicerata	489	497–479	Scorpiones	354	395–300
Arachnida	485	494–475	Amblypygi	97	114–82
Arachnopulmonata	473	483–461	Araneae	352	375–328

The age of the Euarthropoda root, given the taxonomic sample, is recovered near the end of the Ediacaran, 546 million years ago (Ma), with the 95% highest posterior density (HPD) lying between 551 and 536 Ma. Chelicerata are inferred to originate at 535 Ma (with HPD 540 – 527 Ma), similar to the age retrieved for Mandibulata 535 Ma (539 – 526 Ma). The origin of Myriapoda comprises ages centered on the early Cambrian 516 Ma (524 – 505 Ma) and precedes that of Pancrustacea at 486 Ma (501 – 471 Ma). Hexapods are inferred to be much younger in age than myriapods, ranging through the Late Ordovician to Early Devonian, 422 Ma (448 – 400 Ma).

Arachnid terrestrialization is inferred to date to the Cambrian to Ordovician, crown-group Arachnida having a mean at 485 Ma (494 – 475 Ma). Therefore, our results support a Cambrian or Early Ordovician origin of two of the three main terrestrial arthropod lineages (myriapods and arachnids). The upper limit is consistent with fossil evidence for stem-group Arachnida (the chasmataspidid trackways noted above) but is substantially older than any crown-group fossils. Within Arachnida, rapid cladogenesis then occurred during the 20 million years that followed their origin, with several crown-group supra-ordinal clades becoming established in this time interval. By around 450 Ma, all 10 stem groups leading to extant orders of chelicerates included in this analysis (out of 12 in total – Palpigrada and Schizomida are unsampled) were already established. Further cladogenesis is inferred to have involved a more gradual tempo of evolution, in particular for Arachnopulmonata and Acari, which originated at ∼ 470 Ma but greatly expanded after the start of the Mesozoic (252 Ma to 66 Ma). Our dating suggests that the oldest crown-group arachnid orders are Opiliones and Parasitiformes, with Silurian and Devonian origins, respectively. Crown-group scorpions have a Devonian to Carboniferous origin, with the sampled extant lineages splitting more recently. For Araneae, the crown-group age is centered on the Devonian–Carboniferous boundary, with most extant mygalomorph and araneomorph lineages diversifying after the Jurassic.

In general, these Paleozoic age estimates for deep nodes within the most intensely sampled arachnid orders are similar to those inferred in other recent molecular dating analyses. For example, our estimates for crown-group Araneae is consistent with the Late Devonian (Fernández et al., 2018) or Early Carboniferous dates (Garrison et al., 2016) retrieved in other transcriptome-based analyses; likewise, a Carboniferous mean age for crown-group opistothele spiders is found in each of these studies. Our estimates encompassing a Late Ordovician median age for crown-group Opiliones corresponds to that estimated using tip dating by Sharma and Giribet (2014), whereas node calibration in that study recovered a Silurian median. In the case of Scorpiones, a Late Devonian to Carboniferous origin of the crown group is closely comparable to the date for the same node by Sharma et al. (2018), but older than the strictly Carboniferous ages estimated by Howard et al. (2019). However, in all cases mentioned the credibility intervals substantially overlap, indicating that these independent studies found results that, despite some differences, are not significantly different and corroborate each other. One exception is from a recent phylotranscriptomic study of Pseudoscorpiones, which retrieved an Ordovician to Carboniferous origin for the group (Benavides et al., 2019), significantly older than the Permian ages retrieved here. This may reflect the much more complete taxonomic coverage of pseudoscorpion diversity in the Benavides et al. (2019) analysis than in ours.

To assess whether our joint prior assumptions were driving our posterior estimates, we also ran the analysis under the priors (i.e., we performed analyses without data) and found that the joint priors allowed a wide possible distribution of ages, for the most part encompassing but not enforcing the posteriors (see Supplementary Data Sheet S2). We also performed a molecular clock analysis from a different matrix that resulted in a topology in which Xiphosura was nested within Arachnida, specifically as the sister group of Arachnopulmonata + Pseudoscorpiones. Overall, the result it is in general agreement with the main analysis, with most significant discrepancies concerning the age of Pycnogonida, which encompasses Silurian to Devonian ages, whereas in the main analysis are centered on the Carboniferous (see Supplementary Data Sheet S3).

### Rate of Molecular Evolution and Diversification

**FIGURE 2:**
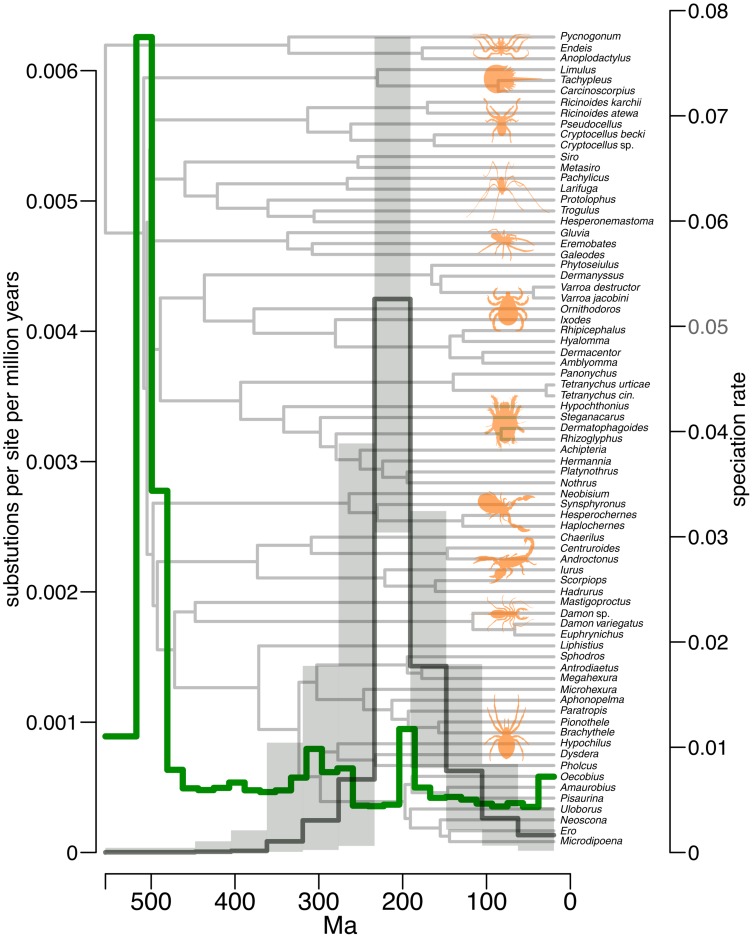
Inferred rates of molecular evolution and diversification over time across chelicerates. Shown on the left *Y* axis are inferred amino acid substitutions per site per million years. On the right *Y* axis are diversification rates estimated with speciation rate as a proxy. Rates of molecular evolution are marked in green. Median speciation rates are marked in black with the highest posterior density of estimates shown in gray. In the timescale, numbers represent millions of years before the present.

We conducted estimations of molecular evolution and diversification rates based on our chelicerate timetree in an attempt to clarify whether the explosive cladogenesis at the onset of the arachnid radiation early in the Phanerozoic was matched by an increase in either of these rates. The analyses of rates of molecular evolution along the branches show a very high rate early in chelicerate history, including at the origin of Euchelicerata (Figure 2). These rates remain high during the early radiation of the Arachnida until the end of the Cambrian. Molecular rate estimations using branch lengths or ancestral sequences under a non-clock model gave nearly identical results. Using the episodic model of speciation and extinction rates through time, we found no evidence of high rates of speciation during the Cambrian, the period that presents the highest rates of molecular evolution. Instead, there is evidence for higher rates of cladogenesis later, bracketed between the Permian and Early Cretaceous, but especially high in the Permian and Triassic (Figure 2).

## Discussion

Molecular clocks allow the reconstruction of evolutionary timescales, but the reliability of these timescales depends on a variety of assumptions, which includes fossil data that have robust stratigraphic and phylogenetic justification, the use of a robust phylogenetic framework for the extant taxa, and the use of well-fitting models of both amino acid substitution and change in rate of molecular evolution. In this context, fossil calibrations then provide minimum ages for the origin of crown groups. Based on current best practice, our analysis uses fossil calibrations following the guidelines set out by Parham et al. (2012) (see Table 1 and Supplementary Data Sheet S4). Furthermore, we report divergence times on a well-sampled phylogeny (Lozano-Fernandez et al., 2019), using the best-fitting molecular substitution and relaxed molecular clock models (see Lozano-Fernandez et al., 2019). We therefore contend that our findings provide the currently most robust insights into early chelicerate evolution (Figure 1). In our analysis, the ancestral pycnogonid divergence from Euchelicerata is inferred to have happened early in the Cambrian. This does not greatly predate the oldest unequivocal total-group pycnogonid, *Cambropycnogon klausmuelleri* (Waloszek and Dunlop, 2002), from the late Cambrian Orsten Konservat-Lagerstätte. The pycnogonid–euchelicerate divergence date suggests cryptic evolution of the euchelicerate stem group in the early Cambrian. Chelicerate – and, indeed, arthropod – body fossils are lacking in the earliest Cambrian, the Fortunian, the arthropod fossil record in the first 20 million years of the Cambrian being limited to trace fossils. Subsequently, it is estimated that xiphosurids diverged from arachnids in the late Cambrian, followed soon after by the radiation of crown-group Arachnida. While revising this paper, a new study on spider fossil calibrations (Magalhaes et al., 2019) came out and suggests that a few of the shallower calibrations used within Araneae treated as crown-groups may instead be stem-groups, so we add this caveat when interpreting the age of spiders.

There remain major geochronological discrepancies between the inferred molecular and fossil age of the various terrestrial arthropod groups. While these discrepancies may be thought to question the accuracy of molecular clocks, the differences need to account for pervasive biases in the terrestrial sedimentary rock record. It has been noted that in Euramerica (from which much of the data on early terrestrial arthropods and early plant megafossils are derived), terrestrial sediments are rare before the late Silurian, and first become widespread in the Early Devonian (Kenrick et al., 2012). This temporal bias in the rock record almost certainly affects the fossil records of terrestrial organisms, and likely accounts for a major component of the discordance between molecular and fossil dates. The common recovery of horseshoe crabs as ingroup arachnids is perhaps unsurprising, given the short molecular branch lengths among these nodes in the tree, which also suggest short divergence times. With a reasonably good xiphosurid and pycnogonid fossil record, the molecular clock is well constrained among euchelicerates. This is evidenced by the short credibility intervals among deep arachnid nodes (Figure 1, Table 2, and Supplementary Data Sheet S1).

From an ecological context, it has been suggested that appreciably complex terrestrial ecosystems may have existed as far back as 1 billion years ago (Clarke et al., 2011), with molecular dating suggesting that crown-group land plants were already present by the middle Cambrian (Morris et al., 2018b). If it is indeed the case that myriapods and arachnids were on land so early, we speculate that the animals may have been early grazers on littoral bacterial mats, or predated on other amphibious or terrestrial organisms (Clarke et al., 2011). These ecologies represent habitats highly unfavorable to fossilization, such as high-energy environments characterized by erosion rather than deposition (Parry et al., 2018). It is unsurprising that paleontological insight is thus limited, and inference of the molecular kind as used here becomes more important as an investigative tool.

We estimate that arachnids colonized the land near the Cambrian–Ordovician boundary, and diversified soon after. Rates of molecular evolution were high at the onset of Arachnida, coinciding with rapid cladogenesis. High rates are concentrated on the branches leading to the major clades within Arachnida, representing major morphological and ecological partitions within the group. In unusually large and ancient clades, such as chelicerates, it is expected to find high rates of molecular evolution in their early lineages (Budd and Mann, 2018, 2019), and we retrieved results in agreement with that expectation (Figure 2). In order to avoid biases related to that fact, we imposed on the molecular clock analyses several maxima on the deepest nodes to account for possible overestimations of divergence times. Arachnids are predominantly predators, which must reflect the presence of an already diverse ecosystem, which the slightly older divergence times for myriapods and embryophytes established in the middle Cambrian. We therefore suspect that arachnids primitively represent carnivorous arthropods rather than having adapted to this mode of life convergently several times. The carnivorous centipede (Chilopoda) crown group is separated from other myriapods by a long branch estimated to be much younger in age (Fernández et al., 2018) than the detritivorous and/or fungal-feeding progoneate myriapods, allowing arachnids to be potentially the first carnivorous animals on land with early myriapod lineages as a likely source of prey. Hexapod divergence estimates are generally younger, suggesting a colonization of land no earlier than the Ordovician (Lozano-Fernandez et al., 2016; Schwentner et al., 2017).

There is a clear contrast in evolutionary tempo after the explosive radiation of the Cambrian and Ordovician. More gradual cladogenesis characterizes later Phanerozoic macroevolutionary dynamics of chelicerates, as is seen in the origins of ordinal clades. Our diversification studies reveal an increase in speciation rates bracketed between the Permian and the Early Cretaceous, in the origin of most sub-ordinal clades, with no evidence of higher speciation rates coinciding with the early rapid arachnid cladogenesis. A heightened diversification of spiders during the Cretaceous has previously been detected, suggested to result from the rise of angiosperms, stimulated by a warmer climate that led to the proliferation of spiders’ main prey, insects (Shao and Li, 2018). Interestingly, we did not observe an early burst of diversification at the origin of chelicerates followed by a slowdown toward the present, a statistical bias usually found in large clades that survive to the present, the so-called “push of the past” (Nee et al., 1994). Instead, it seems that speciation rates are decoupled from the rates of molecular change. The common origin of arachnids giving rise to a plethora of adaptations, together with high molecular rates on the short internodes at the origin of the group suggests an ancient adaptive radiation shortly after colonizing the land, but our diversification analyses have not detected higher speciation rates at that time, one of the key features signaling an adaptive radiation (Glor, 2010). We acknowledge that the taxon sampling may not be the most adequate to infer speciation rates, as it was originally designed to maximize diversity, particularly at the deepest nodes to resolve the splits at the ordinal level.

## Conclusion

Our analysis corroborates euchelicerates having radiated in the Cambrian and arachnids having diversified rapidly in the latest Cambrian–Early Ordovician. While this radiation was rather fast (see Figure 1), we found no evidence that the speciation rates that underpinned it were explosive. The late Cambrian to Early Ordovician emergence of arachnid stem groups onto land was soon followed by a rapid radiation near that same geological boundary, cladogenesis coinciding with high rates of molecular evolution during that time. A later phase of diversification within Arachnida is detected between the Permian and Early Cretaceous, during which the living arachnid orders exhibit heightened rates of speciation.

## Data Availability Statement

All datasets generated for this study are included in the article/Supplementary Material.

## Author Contributions

JL-F, AT, DP, GE, and JV designed the experiments. JL-F, GE, and DP authored the main text with further suggestions from all other authors. JL-F carried out the sequencing laboratory work. JL-F, AT, and MP carried out the matrix compilation and computational analyses. AT, JL-F, and MP designed the figures.

## Conflict of Interest

The authors declare that the research was conducted in the absence of any commercial or financial relationships that could be construed as a potential conflict of interest.

## Supplementary Material

DATA SHEET S1 Like Figure 1, but with the outgroups shown.

DATA SHEET S2 Comparison for selected nodes of the 95% credibility intervals of the estimated divergence times generated by the joint priors (in blue) versus the posterior estimates (in red). The circles represent the average ages.

DATA SHEET S3 Chelicerate divergence times in the molecular clock analysis not recovering Arachnida (outgroups shown) Divergence times shown are obtained under the CIR autocorrelated, relaxed molecular clock model. Nodes in the tree represent average divergence times estimated. The density plots represent the posterior distributions from the considered node. The numbered blue circles represent the age of the fossil calibrations and are located at a height corresponding to the node they are calibrating (see Table 1). In the timescale on the *X* axis, numbers represent millions of years before the present, and the geological period noted.

DATA SHEET S4 Fossil justifications.

DATA SHEET S5 Two molecular matrices, the guiding trees and the calibration file used in PhyloBayes analyses with the retrieved chronograms, and the subset of sampled timescaled trees used to generate the posterior distributions shown on the figures. The input files used for the diversification analyses are also included.
