# Supplementary figures and images for: A Cambrian–Ordovician Terrestrialization of Arachnids
Source: Front Genet. 2020 Mar 11;11:182. doi: 10.3389/fgene.2020.00182 (PMC7078165; doi:10.3389/fgene.2020.00182)

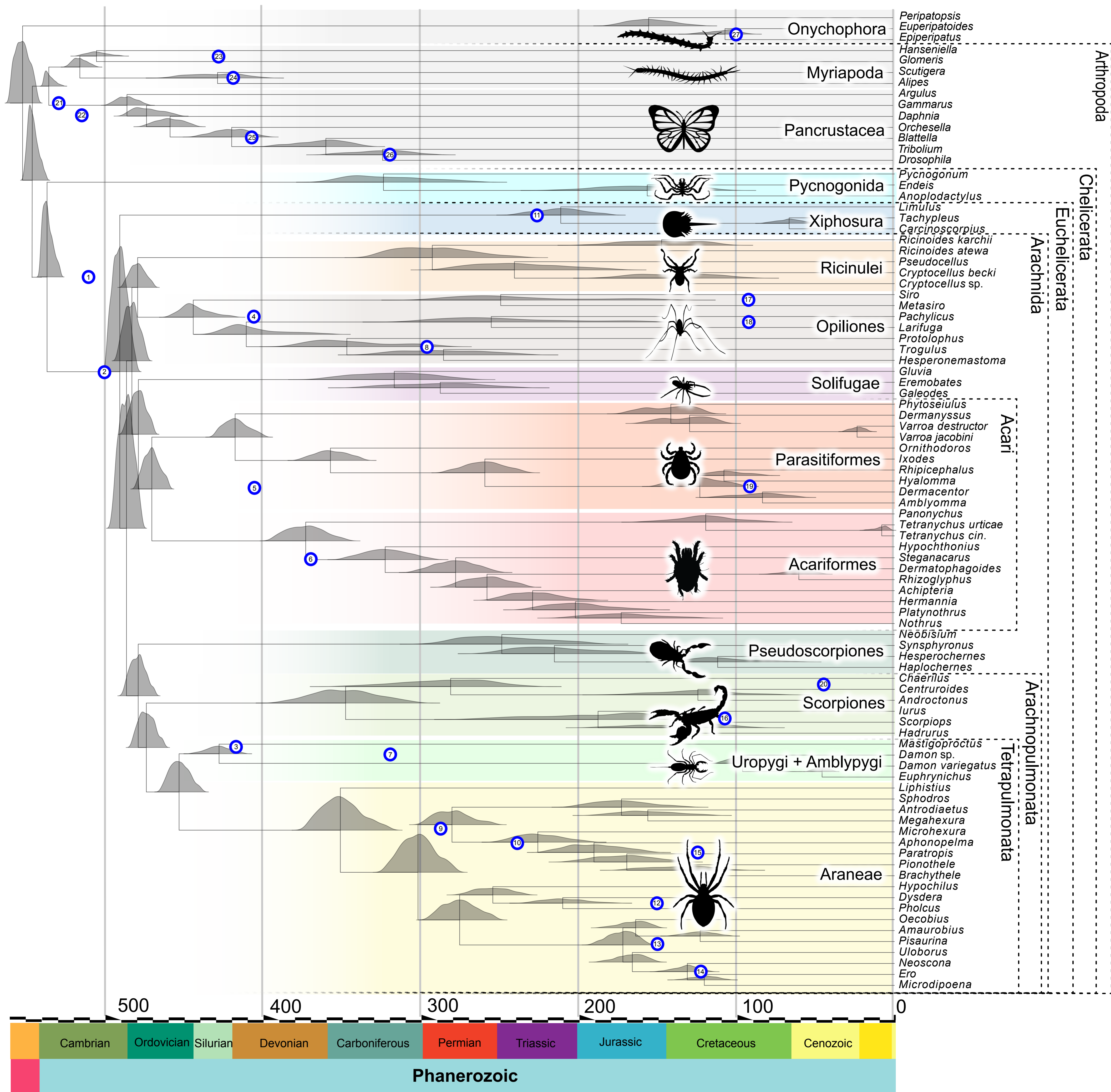

Supplement: DATA SHEET S1 — Like Figure 1, but with the outgroups shown. [file Data_Sheet_1.PDF]

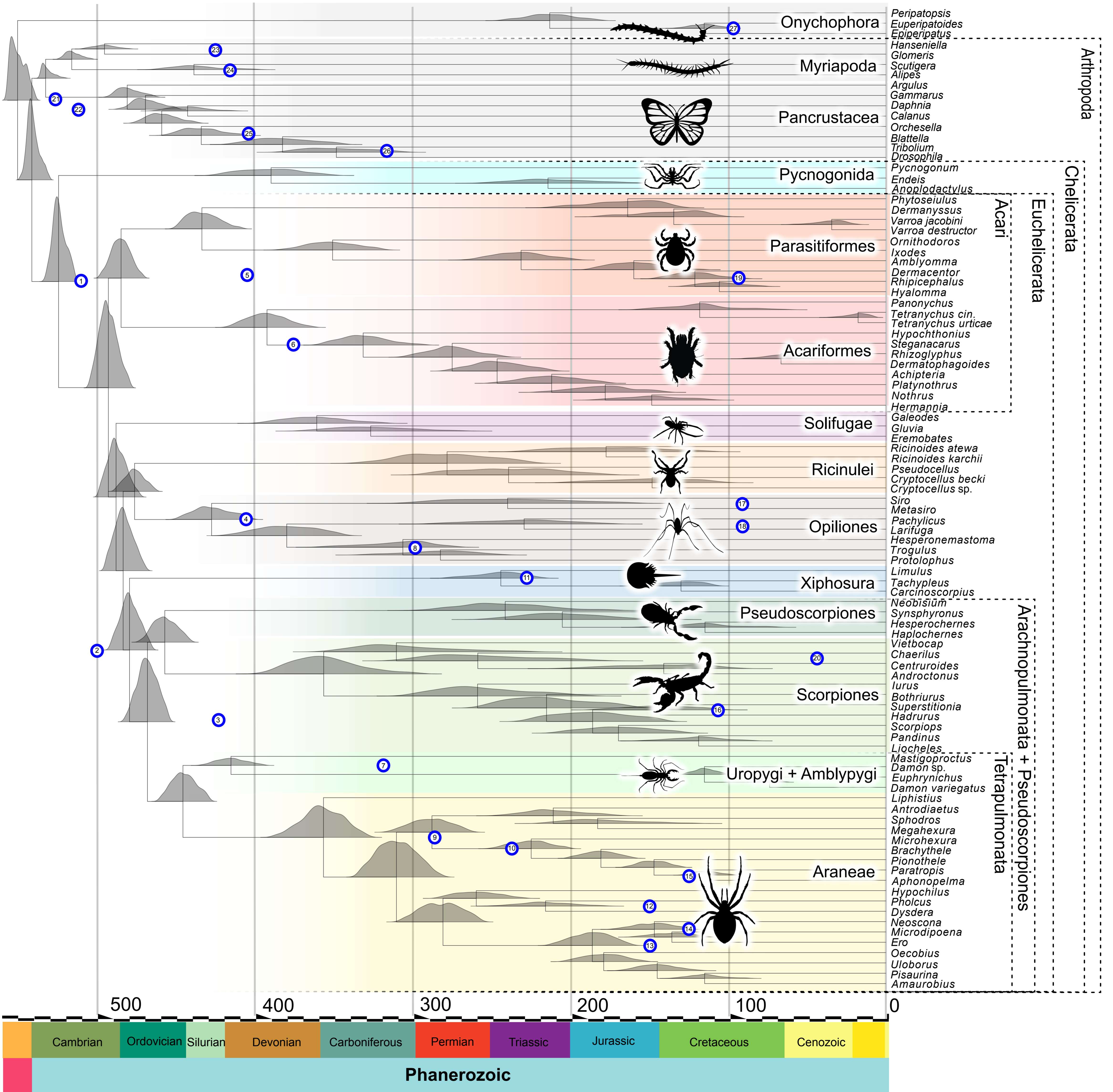

Supplement: DATA SHEET S2 — Comparison for selected nodes of the 95% credibility intervals of the estimated divergence times generated by the joint priors (in blue) versus the posterior estimates (in red). The circles represent the average ages. [file Data_Sheet_2.PDF]

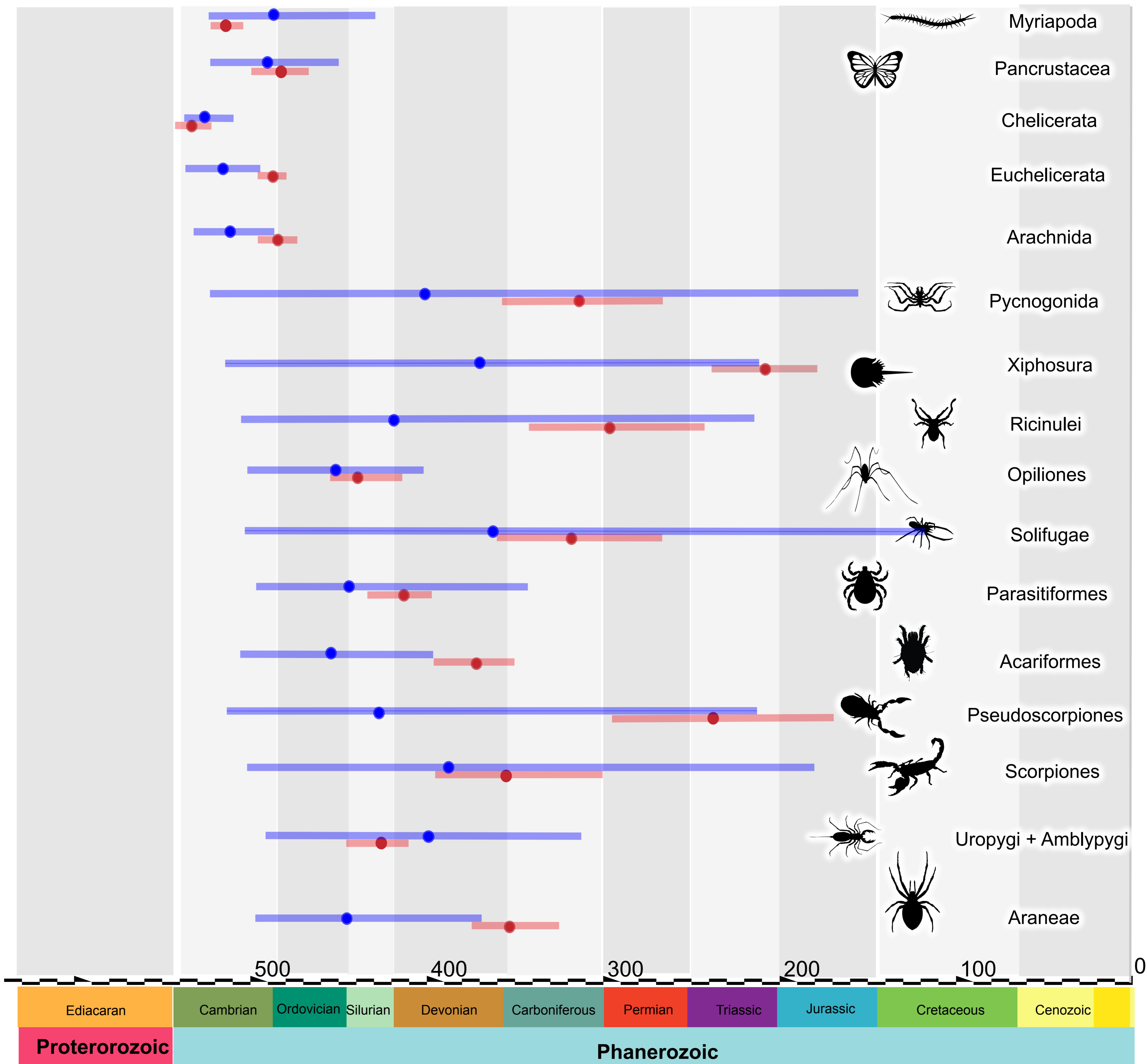

Supplement: DATA SHEET S3 — Chelicerate divergence times in the molecular clock analysis not recovering Arachnida (outgroups shown) Divergence times shown are obtained under the CIR autocorrelated, relaxed molecular clock model. Nodes in the tree represent average divergence times estimated. The density plots represent the posterior distributions from the considered node. The numbered blue circles represent the age of the fossil calibrations and are located at a height corresponding to the node they are calibrating (see Table 1). In the timescale on the X axis, numbers represent millions of years before the present, and the geological period noted. [file Data_Sheet_3.PDF]
