# Supplementary material for: A Cambrian–Ordovician Terrestrialization of Arachnids
Source: Front Genet. 2020 Mar 11;11:182. doi: 10.3389/fgene.2020.00182 (PMC7078165; doi:10.3389/fgene.2020.00182)
Supplement: DATA SHEET S4 — Fossil justifications. [file Data_Sheet_4.PDF]

A Cambrian-Ordovician terrestrialization of arachnids

Authors

Jesus Lozano-Fernandez; Alastair R Tanner; Mark N Puttick; Jakob Vinther; Gregory D Edgecombe; Davide Pisani

Electronic Supplementary Material

FOSSIL CALIBRATIONS

Nodes calibrated by Wolfe et al. (2016) are justified therein, and data for those nodes in Table 1 are as in that work. The taxonomic sampling used in the present analyses allows numerous additional nodes to be calibrated by fossils. As in Wolfe et al. (2016), nodes are defined as crown-groups.

Crown Onychophora

[calibration 27]

This clade comprises Peripatidae and Peripatopsidae, their last common ancestor and all of its descendants. Monophyly is supported by analyses of multilocus sequence data (Giribet et al. 2018).

Fossil specimens

Cretoperipatus burmiticus Grimaldi at al., 2002: holotype, AMNH Bu 218, undetermined sex, anterior part of body in amber, from Tanai Village (on Ledo Road, 105 km NW of Myitkyina), Kachin, Burma. Additional figured specimens assigned to this species by Oliviera et al. (2016).

Phylogenetic justification

Originally assigned to Peripatidae based on its distribution of crural papillae (Grimaldi et al. 2002), more reliable diagnostic characters of that family have been identified in details of the dermal papillae, the diastema in the jaws, and the position of the genital pad, and phylogenetic comparisons have been made with particular extant ingroup peripatid genera (Oliviera et al. 2016). As such *C. burmiticus* is an unambiguous crown-group onychophoran.

Age justification

An early Cenomanian age for Burmese amber follows Wolfe et al. (2016, p. 57), drawing on recent U-Pd dating of zircons from volcaniclastic matrix (Shi et al. 2012), applying a date of 98.17 Ma.

Crown Progoneata

[calibration 23]

This clade comprises Diplopoda (millipedes), Pauropoda and Symphyla, their last common ancestor, and all of its descendants. Monophyly is supported by phylogenetic analysis of nuclear protein-coding genes (Regier et al., 2010; Zwick et al., 2012), whole mitochondrial genomes (Brewer et al., 2013), analyses of transcriptomes (Fernandez et al. 2018), and morphology (Edgecombe, 2004).

Fossil specimens

Casiogrammus ichthyeros Wilson, 2005: holotype NMS (National Museum of Scotland) 1970.2, from the Fish Bed Formation, Glenbuck Group, Smithy Burn, Hagshaw Hills Inlier, Lanarkshire, Scotland, part and counterpart preserved as an articulated series of 19 partly exfoliated trunk segments in siltstone.

Phylogenetic justification

Casiogrammus was originally classified together with a Carboniferous millipede, *Zosterogrammus stichostrethus* Wilson, 2005, in an extinct order, Zosterogrammida, based sharing broad terga with distinctive ornament. The better known *Zosterogrammus* provides most of the relevant data for assigning Zosterogrammida to Chilognatha. Coding *C. ichthyeros* in a morphological dataset recovers it as total-group Chilognatha (crown-group Diplopoda) and thus, for the taxonomic sampling herein, crown-group Progoneata (Fernández et al. 2018: fig. 2e).

Age justification

The Fish Bed Formation receives a Wenlock date based on its spores (Wellman and Richardson 1993). A minimum date using the base of the Ludlow Series is applied, 427.4 Ma ± 0.5 Myr (426.9 Ma).

Crown Acari

[calibration 5]

This clade is composed of Acariformes, Parasitiformes, their last common ancestor and all of its descendants. Monophyly is supported by morphology (extant taxon analysis of Shultz 2007) and analysis of transcriptomes (Lozano-Fernandez et al. 2019).

Fossil specimens

Protacarus crani Hirst, 1923; BMNH In. 24665, holotype, a nearly complete individual in chert.

Phylogenetic justification

The Early Devonian Acariformes of the Rhynie Chert have been of debated systematic position (Hirst 1923; Dubinin 1962), but all assignments to extant families (Alicorhagiidae, Alcynidae, Nanorchestidae) place them within "Endeostigmata" *sensu* Pepato and Klimov (2015), and they are thus crown-group Acariformes but are excluded from a more restricted Sarcoptiformes + Trombidiformes clade that is retrieved in our taxonomic sampling of acariforms. They are accordingly crown-group Acari.

Protacarus crani has been interpreted as a mix of species (Dubinin, 1962). Irrespective of this, the holotype and similar specimens possess fan-like setae dorsally that support a relationship with endeostigmatans (Bernini 1986; Dunlop and Selden 2009).

Additional (but younger) Devonian data for "Endeostigmata" are provided by the Givetian alicorhagiid *Archaeacarus dubinii* Kethley et al., 1989, from Gilboa, New York.

Age justification

Dating of the Rhynie Chert is as summarised by Wolfe et al. (2016, p. 56), a minimum age of 405.0 Ma, using the Pragian-Emsian boundary (407.6 Ma ± 2.6 Myr) as a reference.

Crown Ixodida

This clade is composed of Nuttalliellidae, Argasidae, Ixodidae, their last common ancestor, and all of its descendants. Monophyly is supported by morphology (Lehtinen 1991) and analyses of 18S rRNA and mitochondrial genomes (Mans et al. 2012).

Fossil specimens

Amblyomma birmitum Chitimia-Dobler et al., 2017. See Metastriata below.

Phylogenetic justification

See Metastriata below.

Age justification

See Onychophora above.

Crown Ixodidae

This clade is composed of Prostriata, Metastriata, their last common ancestor and all of its descendants. Monophyly is supported by analyses of 18S rRNA and mitogenomics (Mans et al. 2012).

Fossil specimens

Amblyomma birmitum Chitimia-Dobler et al., 2017. See Metastriata below.

Phylogenetic justification

See Metastriata below.

Age justification

See Onychophora above.

Crown Metastriata

[calibration 19]

This clade is composed of *Amblyomma*, *Rhipicephalus*, their last common ancestor and all of its descendants. Monophyly is supported by analyses of 18S rRNA and mitogenomics (Mans et al. 2012).

Fossil specimens

Amblyomma birmitum Chitimia-Dobler et al., 2017; holotype F24671BN/CJW, Jörg Wunderlich collection, to be deposited in the Senckenberg Museum Frankfurt/Main, an unengorged female in amber, Hukawng Valley, Kachin, Burma.

Phylogenetic justification

Amblyomma biritum was assigned to an extant genus. *Amblyomma* is nested within Metastriata, one of the two clades into which Ixodidae is divided (the other, Prostriata, consists of *Ixodes* alone) (reviewed by Mans et al., 2012; Chitimia-Dobler et al., 2017, 2018). Accordingly *A. birmitum* is a member of crown-group Ixodidae and thus of crown-group Ixodida.

Age justification

See Onychophora above.

Remarks

Three additional species of Ixodidae from Burmese amber, *Cornupalpatum burmanicum* Poinar and Brown, 2003, *Compluriscutata veletum* Poinar and Buckley, 2008, and *Haemaphysalis* (*Alloceraea*) *cretacea* Chitimia-Dobler et al., 2018, provide the same age data and similar phylogenetic data as

Amblyomma birmitum. They are likewise identified as members of Metastriata by Chitimia-Dobler et al. (2017, 2018), *H. cretacea* (assigned to an extant genus and subgenus) being the most confidently referred.

Crown Sarcoptiformes + Trombidiformes

This clade comprises all Acariformes to the exclusion of "Endeostigmata". Monophyly is supported by analyses of nuclear ribosomal genes (Pepato and Klimov 2015).

Fossil specimens

Protochthonius gilboa Norton et al., 1988. See Sarcoptiformes below.

Phylogenetic justification

See Sarcoptiformes below.

Age justification

See Sarcoptiformes below.

Remarks

The earliest crown-group members of Trombidiformes are the Late Triassic *Triasacarus fedelei* and *Ampezzoa triassica* Lindquist and Grimaldi (in Schmidt et al., 2012), and two additional monotypic genera from the same Dolomite amber described by Sidorchuk et al. (2014). These do not provide a crown-group calibration for this study because none nests within the extant Trombidiformes sampled herein.

Crown Sarcoptiformes

[calibration 6]

This clade is composed of Oribatida (paraphyletic), Astigmata, their last common ancestor and all of its descendants. Monophyly is supported by analyses of nuclear ribosomal genes (Pepato and Klimov 2015) and a six-gene compilation (Klimov et al., 2018).

Fossil specimens

Protochthonius gilboa Norton et al., 1988; holotype AMNH 43117 (411-7-AR-38 of Norton et al. 1988), a largely complete slide-mounted specimen.

Phylogenetic justification

P. gilboa is assigned to an extinct family, Protochthoniidae Norton et al., 1988, but belongs to the Enarthronota within the Oribatida (Norton et al. 1988; Subias and Arillo 2002). This is based on hypertrophied, erectile setae with *e* and *f* rows, and undivided femora that are apomorphic for Enarthronota (Norton et al. 1988). It is accordingly crown group Sarcoptiformes and thus within the crown group of the more inclusive clade that unites Sarcoptiformes and Trombidiformes.

Age justification

P. gilboa is from the upper part of the Panther Mountain Formation at Gilboa, Schoharie County, New York State. It is dated to the Tioughniogan Regional Stage, Givetian in the global time scale.

Palynomorphs are consistent with a Givetian age (Richardson et al. 1993). Accordingly, a minimum date for the end of the Givetian/base of the Frasnian is applied (382.7 Ma).

Remarks

The same calibration is provided by other crown-group Sarcoptiformes from the Gilboa locality, such as *Ctenacaronychus nortoni* Subias and Arillo, 2002, assigned to the oribatid suborder Palaeosomata.

Crown Cyphophthalmi

[calibration 17]

This clade is composed of Scopulophthalmi, Sternophthalmi, “Boreophthalmi” (all *sensu* Giribet et al. 2012), their last common ancestor and all of its descendants. Monophyly is supported by morphology and its combination with multi-locus sequence data (Giribet et al. 2010, 2012).

Fossil specimens

Palaeosiro burmanicum Poinar, 2008; holotype, complete specimen in amber, deposited in the Poinar amber collection (accession #B-1-17), maintained at Oregon State University, Corvallis.

Phylogenetic justification

Palaeosiro was originally assigned to the family Sironidae (Poinar 2008) but was subsequently transferred to the family Stylocellidae by Giribet et al. (2012) based on the shape and position of the ozophores, the presence of eyes, the carina on the anal plate, and the geographic distribution in Southeast Asia. Either assignment places it within crown-group Cyphophthalmi (phylogeny from Giribet et al. 2010, 2012; Fernández et al. 2017), the crown-group of Sternophthalmi + Boreophthalmii (=all Cyphophthalmi to the exclusion of Pettalidae), and crown-group Boreophthalmi (=Sironidae + Stylocellidae).

Age justification

P. burmanicum is from Burmese amber at a mine in the Hukawng Valley, southwest of Maingkhwan, Kachin. Dating is as for Onychophora above.

Crown Phalangida

This clade is composed of Eupnoi, Laniatores and Dyspnoi, their last common ancestor and all of its descendants. Monophyly is supported by morphology (Shultz 1998, Giribet et al. 2002), combined molecules and morphology (Giribet et al. 2002; Garwood et al. 2011, 2014), and transcriptomes (Hedin et al. 2012; Fernández et al. 2017).

Fossil specimens

Macrogyrion cronus Garwood et al., 2011. See Palpatores below.

Phylogenetic justification

See Palpatores below.

Age justification

See Palpatores below.

Crown Palpatores

[calibration 8]

This clade is composed of Dyspnoi, Eupnoi, their last common ancestor, and all of its descendants. Monophyly is supported by morphology (Shultz 1998), combined molecules and morphology (Garwood et al. 2014; Sharma and Giribet 2014), and transcriptomes (Hedin et al. 2012; Sharma and Giribet 2014; Fernández et al. 2017).

Fossil specimens

Macrogyrion cronus Garwood et al., 2011; holotype MNHN-SOT 079398 (Collection Sotty 2, deposited in the Muséum d'Histoire naturelle d'Autun), belonging to the Museum national d'Histoire naturelle, Paris (MNHN 079398), a nearly complete specimen preserved as counterpart moulds in a siderite nodule.

Phylogenetic justification

Phylogenetic analysis in its original description resolved *Macrogyrion* within total-group Eupnoi (Garwood et al. 2011) based on parsimony analysis of morphological characters and Bayesian inference based on combined morphological and molecular data, and this position is congruent with subsequent analyses that included additional fossils and different taxon samples, although these more explicitly resolved it as stem-group Eupnoi (Garwood et al. 2014; Sharma and Giribet 2014). It is accordingly crown-group Phalangida and crown-group Palpatores.

Age justification

The Montceau-les-Mines Konservat-Lagerstätte in central France, from which *M. cronus* derives, is dated to Stephanian B/C, correlative with the Kasimovian/Gzhelian boundary interval. Following Wolfe et al. (2016, p. 60), we date based on the upper boundary of the Gzehelian for a minimum of 298.75 Ma.

Remarks

Ameticos scolos Garwood et al. 2011, also from the Montceau-les-Mines Lagerstätte, was resolved as total-group Dyspnoi based on the same morphological and molecular datasets that resolved *Macrogyrion* as total-group Eupnoi (Garwood et al. 2011). Subsequently it has been resolved within as total-group Dyspnoi (Garwood et al. 2014; Sharma and Giribet 2014). It therefore provides the same age constraints for Phalangida and Palpatores as does *Macrogyrion cronus*.

Crown Laniatores

[calibration 18]

This clade comprises Travunoidea and Grassatores, their last common ancestor, and all of its descendants. Monophyly is indicated by morphology (Giribet et al. 2002), combination of morphology and multi-locus sequence data (Giribet et al. 2010), and transcriptomes (Sharma and Giribet 2014; Fernández et al. 2017).

Fossil specimens

Petrobunoides sharmai Selden et al., 2016; holotype BU-002036, currently housed in the Key Lab of Insect Evolution and Environmental Changes, College of Life Sciences, Capital Normal University, Beijing, for eventual deposition in Three Gorges Entomological Museum, Chongqing.

Phylogenetic justification

P. sharmai demonstrates apomorphic characters of Laniatores, such as the scutum magnum condition (carapace and first five opisthosomal tergites fused into a single dorsal plate) and strongly raptorial pedipalps (Selden et al. 2016). It is more precisely assigned to one of the two Laniatores infraorders, Grassatores, based on the apomorphy of two tarsal claws on legs III and IV, and is accordingly a member of crown-group Laniatores. Membership in the crown group of Grassatores (and thus corroboration for the two more general nodes above) is indicated by its assignment to the extant Grassatores family Epedanidae based on the relatively long chelicera with the proximal segment unadorned on the dorsal surface, the elongate raptorial pedipalps, the elongate gracile legs with a tarsal formula consistent with Epedanidae, and the wide ocularium and subquadrilateral body (Selden et al. 2016).

Age justification

The holotype and sole known specimen is from Burmese amber. The type locality is a mine on Noije Bum hill, near Tanai Village in the Hukawng Valley. The age of the Cretaceous Burmese amber is discussed above under Ixodida.

Crown Opisthothelae

This clade is composed of Mygalomorphae, Araneomorphae, their most recent common ancestor and all of its descendants. Monophyly is supported by morphology (Coddington 2005) and analyses of transcriptomic datasets (Bond et al. 2014; Garrison et al. 2016; Fernández et al. 2018).

Fossil specimens

Rosamygale grauvogeli Selden and Gall, 1992. See Avicularoidea below.

Phylogenetic justification

See Avicularoidea below.

Age justification

See Avicularoidea below.

Crown Mygalomorphae

This clade is composed of Atypodidea, Avicularoidea (both sensu Goloboff 1993), their last common ancestor and all of its descendants. Monophyly is supported by morphology (Raven 1985; Coddington 2005), morphological data combined with nuclear genes (Bond et al. 2012), and transcriptomics (Bond et al. 2014; Garrison et al. 2016; Fernández et al. 2018).

Fossil specimens

Rosamygale grauvogeli Selden and Gall, 1992. See Avicularoidea below.

Phylogenetic justification

See Avicularoidea below.

Age justification

See Avicularoidea below.

Crown Avicularoidea

[calibration 10]

This clade is composed of "Dipluridoids", Hexathelidae, Bipectina, their last common ancestor, and all of its descendants. Monophyly is supported by morphology (Goloboff 1993) and transcriptomics (Bond et al. 2014).

Fossil specimens

Rosamygale grauvogeli Selden and Gall, 1992; holotype AR-11, part and counterpart, Gravogel-Gall collection, Institut de Geologie, Université Louis Pasteur, Strasbourg, from Grès à Voltzia, Vosges, France.

Phylogenetic justification

Rosamygale is confidently attributed to Mygalomorphae based on the diagnostic presence of six spinnerets that are posteriorly situated on the abdomen, the posterior lateral spinnerets (PLS) being composed of three segments, and paraxial chelicerae (Selden and Gall 1992), drawing on diagnostic characters of Raven (1985). It was tentatively assigned to the extant avicularoid family Hexathelidae in its original description (Selden and Gall 1992), exhibiting characters of a broader Tuberculotae (sensu Raven 1985), to which hexathelids were assigned. Monophyly of Hexathelidae was subsequently rejected by Bond et al. (2012) based on molecular data, as anticipated by Goloboff (1993), and "Tuberculotae" likewise discarded, its characters being symplesiomorphies for Avicularoidea (Goloboff 1993). Additional support for hexathelid affinities was noted by Selden (2002), spigots occurring along the length of the PLS. Magalhaes et al. (2019) suggest that an assignment of *Rosamygale* to the stem (rather than crown) of Avicularoidea is justified, but accept an attribution to crown-group Mygalomorphae.

Age justification

The specimens are derived from a clay facies in the Grès à Meule unit in the lowermost part of the Grès-à-Voltzia Formation. Following Wolfe et al. (2016, p. 84), sequence stratigraphy dates this level to the middle Anisian, applying a minimum age for the top of the Anisian of 240.5 Ma.

Crown Bipectina

[calibration 15]

This clade is composed of Diplurinae and Rastelloidina + Crassitarsae, their last common ancestor, and all of its descendants. Monophyly is supported by morphology (Goloboff 1993), combination of morphology and three nuclear genes (Bond et al. 2012), and transcriptomes (Bond et al. 2014).

Fossil specimens

Cretamygale chasei Selden, 2002; holotype IWCMS.1994.101, a largely complete female or juvenile in amber from lignitic marls between the Chilton Chine Sandstone and Brighstone Sandstone in the Wessex Formation, Isle of Wight, UK.

Phylogenetic justification

Tentatively assigned to the extant family Nemesiidae, *C. chasei* is compatible with the crown-group of the clade Bipectina based on the following characters listed by Selden (2002): eyes grouped on a tubercle anteromedially on the carapace; the saddled area of the carapace not being strongly raised; a sloping thoracic part of the carapace; a deep, transverse fovea; a pair or large, erect setae on the

anterior wall of the fovea; symmetrically arranged tarsal/metatarsal scapulae on leg 1; tarsus of leg 1 cracked ventrally; no claw tufts on leg 1. Magalhaes et al. (2019) recommend an assignment no more precise than to Avicularoidea.

Age justification

The amber-bearing marls in the Wessex Formation are dated as early Barremian (Hughes and McDougall 1990). The top of the Barremian is dated at 125.0 Ma, providing a minimum age.

Crown Araneomorphae

This clade is composed of Hypochilidae, Filisatidae, Synspermiata, Entelegyne, their last common ancestor and all of its descendants. Morphology is supported by morphology (Coddington 2005) and analyses of transcriptomes (Bond et al. 2014; Fernández et al. 2014, 2018; Garrison et al. 2016).

Fossil specimens

Eoplectreurys gertschi Selden and Huang, 2010. See Synspermiata below.

Phylogenetic justification

See Synspermiata below.

Age justification

See Synspermiata below.

Remarks

Two total-group araneomorph taxa are known from the Upper Triassic (Norian), *Triassaraneus andersonorum* Selden in Selden et al., 1999, from the Molteno Formation in South Africa, and *Argyrarachne solitus* Selden in Selden et al., 1999, from the Cow Branch Formation in Virginia, USA. Although both have been identified as possible Araneoidea (Selden et al., 1999), they have most recently been depicted as stem-group Araneomorphae (Selden et al. 2009; Selden and Penney 2010, fig. 2), rather than nested in the araneomorph crown group. Accordingly we have not used them to constrain any crown group nodes within Araneomorpha (e.g. Entelegynae).

Other Araneomorphae from the Daohugou Beds provide the same age data as does *Eoplectreurys gertschi. E. gertschi* is selected for calibration because it permits the most precise systematic assignment.

Crown Synspermiata

[calibration 12]

This clade, named by Michaelik and Ramírez (2014), is composed of Dysderoidea, Scytodoidea, the Lost Tracheae Clade, their last common ancestor, and all of its descendants. Monophyly is supported the fusion of several spermatids into one synsperm, by multi-locus sequence data (Wheeler et al. 2017) and analyses of transcriptomes (Fernández et al. 2018).

Fossil specimens

Eoplectreurys gertschi Selden and Huang, 2010; holotype NIGP 151720a, b, adult male, part and counterpart in finely laminated pale grey tuff, Jiulongshan Formation, Daohugou Village, Wuhua Township, Ningcheng County, Inner Mongolia, northeastern China.

Phylogenetic justification

Described as the oldest haplogyne spider, *E. gertschi* was assigned to the extant family Plectreuridae because it is a short-legged, ecribellate “haplogyne” with fused chelicerae that exhibits close similarities to modern plectreurid genera. Additional characters that were noted to be comparable to Plectreuridae are: tubercles with setae on chelicera, denticles on cheliceral margin, male with strong leg 1 femur and tibia, tibia 1 with large macrosetae (spines) on basal tubercles in distal retrolateral position (clasping spur), macrosetae around distal joint of metatarsus, three-clawed tarsus, and simple palpal bulb with very long embolus sharply set off from bulb (Selden and Huang 2010). Affinities to Plectreuridae render it crown-group Synspermiata, and accordingly crown-group Araneomorphae. Affinities to Plectreuridae nest it within the extant taxa of Synspermiata sampled here (Plectreuridae is more closely related to Pholcidae than to Dysderidae; morphology-based phylogeny from Coddington 2005; multi-locus molecular tree of Wheeler et al. 2017, fig. 3). The compendium of fossil calibrations for spiders by Magalhaes et al (2019), published after these analyses were completed, treats *E. gertschi* as a stem- rather than crown-group member of Synspermiata.

Age justification

The age of the Daohugou beds was reviewed by Wolfe et al. (2016: 89), the youngest available radiometric date being Late Jurassic (Oxfordian). Following that work, we apply a minimum age of 158.1 Ma.

Crown Entelegynae

[calibration 13]

This clade is composed of Eresoidea, Palpanimoidea and a Canoe Tapetum Clade (Coddington 2005), their last common ancestor and all of its descendants. Monophyly is corroborated by morphology (Griswold et al., 2005) and analyses of transcriptomes (Garrison et al. 2016).

Fossil specimens

Mongolarachne jurassica (Selden et al., 2011); holotype female, CNU-ARA-NN2010008, College of Life Sciences, Capital Normal University, Beijing. Male described by Selden et al. (2013).

Phylogenetic justification

Originally described as a species of the extant genus *Nephila* based on the female, the associated male shows distinctive palps that prompted assignment to a new genus and monotypic family, Mongolarachnidae (Selden et al. 2013). It is demonstrably cribellate, though as a plesiomorphic character for Arananeomorphae, this does not provide a positive basis for taxonomic assignment. Although it shows a substantial overall similarity to Orbiculariae, constraining key characters against a backbone tree for relationships of extant araneomorphs is inconsistent with a position within the crown of Orbiculariae or its consistent taxa (Deinopoidea and Araneoidea) (Selden et al. 2013, fig. 5). It may be a stem-group orbicularian but until it is more firmly placed phylogenetically, it is used only to constrain the age of Entelegynae.

Magalhaes et al (2019), published after these analyses were completed, do not regard *M. jurassica* to have sufficiently diagnostic apomorphies to serve for calibration (its putative epigynum does not demonstrably have copulatory openings distinct from the epigastric furrow), and constrain Entelegynae only to the Early Cretaceous, indicating *Eocoddingtonia eskoi* Selden as a stem-group constraint for the clade.

Age justification

See Araneomorphae above.

Remarks

Juraraneus rasnitsyni Eskov, 1984, is another cribellate araneomorph from the Jurassic that has been associated with Orbiculariae, and originally interpreted as an araneoid. Restudy of the holotype and only known specimen (Selden 2012) reinforced comparison to araneoids, but a preliminary phylogenetic analysis (Selden et al. 2013) is consistent with affinities to Entelegynae without establishing a more precise assignment. Its age is Oxfordian (Late Jurassic), thus not demonstrably older than *Mongolarachne* from the Daohugou Beds.

Crown Araneoidea

[calibration 14]

This clade unites entelygyne spiders that use adhesive threads in the webs. Monophyly is supported by analyses of transcriptomes (Garrison et al. 2016; Fernández et al. 2018).

Fossil specimens

Unnamed Linyphiinae from Lebanese amber (Penney and Selden 2002). Specimen No. 491, Laboratoire d'Entomologie, Muséum National d'Histoire Naturelle, Paris, female in amber, from Kdeirji/Hammana outcrop, Lebanon.

Phylogenetic justification

Systematic attribution follows the assignment of the species to Linyphiinae (Penney and Selden 2002). Maghalaes et al. (2019) only tentatively consider this fossil to be a stem-group araneoid, and nominate the slightly younger *Mesozygiella dunlopi* Penny and Ortunio (2006) from the Alava amber (Aptian-Middle Albian) as the oldest unambiguous araneoid.

Age justification

Dating of Lebanese amber follows Wolfe et al (2016, p. 63), using a minimum date of 129.41 Ma.

Crown Iurida

This clade comprises Iuroidea, Bothriuroidea, Scorpionoidea, "Chactoidea" (all sensu Sharma et al. 2015), their last common ancestor, and all of its descendants. Monophyly is supported by analyses of transcriptomes (Sharma et al. 2015, 2018).

Fossil specimens

Protoischnurus axelrodurum Carvalho and Lourenço, 2001. See Bothriuoidea + Scorpionoidea + "Chactoidea" below.

Phylogenetic justification

See Bothriuoidea + Scorpionoidea + "Chactoidea" below.

Age justification

See Bothriuoidea + Scorpionoidea + "Chactoidea" below.

Remarks

Araripescorpius ligabuei Campos, 1986, was revised as a member of Chactidae by Menon (2007), providing the same temporal constraint for Iurida and its subclade Bothriuroidea + Scorpionoidea + "Chactoidea" as does the better known *Protoischurus axelrodurum*.

Crown Bothriuroidea + Scorpionoidea + "Chachtoidea"

[calibration 16]

Monophyly of this clade (superfamilies sensu Sharma et al. 2015) is supported by analyses based on transcriptomes (Sharma et al. 2015, 2018).

Fossil specimens

Protoischnurus axelrodurum Carvalho and Lourenço, 2001. MN-7601-I, holotype, part and counterpart, a male, from the Santana Group, Crato Formation, Ceará, Crato area, Brazil. This specimen was destroyed in the fire at the Museu Nacional, Rio de Janeiro, but a colour photograph of it has recently been published (Howard et al. 2019, fig. 5d). We also refer to the specimen SMNS 65534, which preserves phylogenetically relevant details of the pedipalps (Fig. 3c in Menon 2007).

Phylogenetic justification

Menon (2007) placed *P. axelrodurum* in Hemiscorpiidae based on, among other characters, an inverse Y-shaped sulcus on the prosoma, the placement of Est trichobothria on the pedipalp chela, and the placement of carinae V2 and V3 on the pedipalp chela, all of which are diagnostic of the hemiscorpidid subfamily Hormurinae (Soleglad et al. 2005). Hemiscorpiidae is classified within Scorpionoidea, and accordingly is a crown-group member of the more inclusive clades Iurida and the subgroup therein that unites Bothriuroidea, Scorpionoidea and "Chactoidea" ((Sharma et al. 2015).

Age justification

The Crato Formation is dated to the Aptian based on palynomorphs, and we follow Wolfe et al. (2016, p. 58) in dating the top of the Aptian to a minimum of 112.6 Ma.

Crown Buthida

[calibration 20]

This clade comprises Buthidae, Chaerilidae, Pseudochactidae, their last common ancestor, and all its descendants. Monophyly is supported by analyses of transcriptomes (Sharma et al. 2015, 2018).

Fossil specimens

Uintascorpio halandrasorum Perry, 1995; holotype, Denver Museum of Nature and Science (DMNH 6004), a complete specimen in shale. Redescribed and reillustrated by Santiago-Blay et al. (2004).

Phylogenetic justification

Originally assigned tentatively to Vaejovidae (Perry 1995), revision of *U. halandrasorum* by Santiago-Blay et al. (2004) defended its membership in the family Buthidae. Apomorphic characters of buthids observed in the fossil include elongated leg coxae IV, absence of lateral carinae on metasomal segment, and presence of pedipalp patellar *DMc* carina (Santiago-Blay et al. 2004). Accordingly *U. halandrasorum* constrains crown-group Buthida and Buthidae + Pseudochactidae.

Age justification

U. halandrasorum derives from the Parachute Creek Member of the Green River Formation, Rio Blanco County, Colorado. Although the formation itself spans a considerable extent of the lower and middle Eocene, a radiometric date for the Parachute Creek Member is 48.66+/- 0.12 Ma (Smith et al. 2003). Accordingly, we apply a date of 48.54 Ma.

Remarks

A few Mesozoic orthosterns that have been attributed to Buthida, notably the Triassic Protobuthus elegans Lourenço and Gall, 2004 (Bundsandstein, France) and the Cretaceous Archeobuthus estephani Lourenço, 2001 (Lebanese amber) and Palaeoburmesebuthus grimaldii Lourenço, 2002 (Burmese amber). All of these were dismissed as crown-group Buthidae/Buthida and indeed even crown-group Scorpiones (Baptista et al. 2006), although additional material of Palaeoburmesebuthidae strengthens the case for a buthid-type trichobothria distribution in that group from Burmese amber (Lourenço 2016). Unchallenged crown-group Buthida first appear in the Eocene.

References

Baptista, C., Santiago-Blay, J.A., Soleglad, M.E. and Fet, V. 2006. The Cretaceous scorpion genus, *Archaeobuthus*, revisited (Scopiones: Archaeobuthidae). Euscorpius, 2006, 35, 1-40.

Bernini, F. 1986. Current ideas on the phylogeny and adaptive radiations of Acarida. Bolletino Zoologico, 53, 279-313.

Bond, J.E., Hendrixson, B.E., Hamilton, C.A. and Hedin, M. 2012. A reconsideration of the classification of the spider Infraorder Mygalomorphae (Arachnida: Araneae) based on three nuclear genes and morphology. PLoS ONE, 7, 6, e38753.

Bond, J.E., Garrison, N.J., Hamilton, C.A., Godwin, R.L., Hedin, M. and Agnarsson, I. 2014. Phylogenomics resolves a spider backbone phylogeny and rejects a prevailing paradigm for orb web evolution. Current Biology, 24, 1765-1771.

Brewer, M.S., Swafford, L., Spruill, C.L. and Bond, J.E. 2013. Arthropod phylogenetics in light of three novel millipede (Myriapoda: Diplopoda) mitochondrial genomes with comments on the appropriateness of mitochondrial genome sequence data for inferring deep level relationships. PLoS ONE, 8, e68005.

Carvalho, M.G.P. and Lourenço, W.R. 2001. A new family of fossil scorpions from the Early Cretaceous of Brazil. Comptes Rendues de l'Académie des Sciences, Paris, Sciences de la Terre et des planètes, 332, 711-716.

Chitimia-Dobler, L., Cancian de Araujo, B., Ruthensteiner, B., Pfeffer, T. and Dunlop, J.A. 2017. *Amblyomma birimitum* a new species of hard tick in Burmese amber. Parasitology, 144, 1441-1448.

Chitimia-Dobler, L., Pfeffer, T. and Dunlop, J.A. 2018. *Haemaphysalis cretacea* a nymph of a new species of hard tick in Burmese amber. Parasitology, 145, 1440-1451.

Coddington, J.A. 2005. Phylogeny and classification of spiders. In: Ubick, D., Paquin, R., Cushing, P.E. and Roth, V. (eds), Spiders of North America: an identification manual. American Arachnological Society, 18-24.

Dubinin, V.B. 1962. Class Acaromorpha: mites, or gnathosomic chelicerate arthropods. In: Rodendorf, B.B. (ed.), Fundamentals of Palaeontology. Academy of Sciences of USSR, Moscow, 447-473.

Dunlop, J.A. and Selden, P.A. 2009. Calibrating the chelicerate clock: a palaeontological reply to Jeyaprakash and Hoy. Experimental and Applied Acarology, 48, 183-197.

Edgecombe, G.D. 2004. Morphological data, extant Myriapoda, and the myriapod stem-group. Contributions to Zoology, 73, 267–252.

Fernández, R., Hormiga, G. and Giribet, G. 2014. Phylogenomic analysis of spiders reveals nonmonophyly of orb weavers. Current Biology, 24, 1772-1777.

Fernández, R., Kallal, R.J., Dimitrov, D., Ballasteros, J.A., Arnedo, M.A. and Giribet, G. 2018. Phylogenomics, diversification dynamics, and comparative transcriptomics across the spider tree of life. Current Biology, 18, 1-9.

Fernández, R., Sharma, P.P., Tourinho, A.L., and Giribet, G. 2017. The Opiliones tree of life: shedding light on harvestmen relationships through transcriptomics. Proceedings of the Royal Society, B, 282, 20162340.

Fernández, R., Edgecombe, G.D. and Giribet, G. 2018. Phylogenomics illuminates the backbone of the Myriapoda Tree of Life and reconciles morphological and molecular phylogenies. Scientific Reports, 8, 83.

Garrison, N.L., Rodriguez, J., Agnarsson, I., Coddington, J.A., Griswold, C.E., Hamilton, C.A., Hedin, M., Kocot, K.M., Ledford, J.M. and Bond, J.E. 2016. Spider phylogenomics: untangling the Spider Tree of Life. PeerJ 4:e1719; DOI 10.7717/peerj.1719.

Garwood, R.J., Dunlop, J.A., Giribet, G. and Sutton, M.D. 2011. Anatomically modern Carboniferous harvestmen demonstrate early cladogenesis and stability in Opiliones. Nature Communications, 2, 444.

Garwood, R.J., Sharma, P.P., Dunlop, J.A. and Giribet, G. 2014. A Paleozoic stem group to mite harvestmen revealed through integration of phylogenetics and development. Current Biology, 24, 1-7.

Giribet, G., Buckman-Young, R.S., Sampaio Costa, C., Baker, C.M., Benavides, L. R., Branstetter, M.G., Daniels, S.R. and Pinto-da-Rocha, R. 2018. The '*Peripatos*' in Eurogondwana? - Lack of evidence that south-east Asian onychophorans walked through Europe. Invertebrate Systematics, 32, 842-865.

Giribet, G., Edgecombe, G.D., Wheeler, W.C. and Babbitt, C. 2002. Phylogeny and systematics of Opiliones: a combined analysis of chelicerate relationships using morphological and molecular data. Cladistics, 18, 5-70.

Giribet, G., Sharma, P.P., Benavides, L.R., Boyer, S.L., Clouse, R.M., de Bivort, B.L., Dimitrov, D., Kawauchi, G., Murienne, J. and Schweninger, P.J. 2012. Evolutionary and biogeographical history of an ancient and global group of arachnids (Arachnida: Opiliones: Cyphophthalmii) with a new taxonomic arrangement. Biological Journal of the Linnean Society, 105, 92-130.

Giribet, G., Vogt, L., González, A.B., Sharma, P. and Kury, A.B. 2010. A multilocus approach to harvestmen (Arachnida: Opiliones) phylogeny with emphasis on biogeography and the systematics of Laniatores. Cladistics, 26, 408-437.

Goloboff, P.A. 1993. A reanalysis of mygalomorph spider families (Araneae). American Museum Novitates, 3057, 1-32.

Grimaldi, D.A., Engel, M.S., Nascimbene, P.S. 2002. Fossiliferous Cretaceous amber from Myanmar (Burma): its rediscovery, biotic diversity, and paleontological significance. American Museum Novitates, 3361, 1-71.

Griswold, C.E., Ramírez, M., Coddington, J.A. and Platnick, N. 2005. Atlas of phylogenetic data for entelegyne spiders (Araneae: Araneomorphae: Entelegynae), with comments on their phylogeny. Proceedings of the California Academy of Sciences, 56, 1-324.

Hedin, M., Starrett, J., Akhter, S., Schönhofer, A.K. and Shultz, J.W. 2012. Phylogenetic resolution of Paleozoic divergences in harvestmen (Arachnida, Opiliones) via analysis of Next-Generation transcriptome data. PLoS ONE, 7, 8,e42888.

Hirst, S. 1923. On some arachnid remains from the Old Red Sandstone. Annals and Magazine of Natural History, 9, 12, 455-474.

Howard, R.J., Edgecombe, G.D., Legg, D.A., Pisani, D. and Lozano-Fernandez, J. 2019. Exploring the evolution and terrestrialization of scorpions (Arachnida: Scorpiones) with rocks and clocks. Organisms, Diversity & Evolution, 19, 71-86.

Hughes, N.F. and McDougall, A.B. 1990. New Wealden correlation for the Wessex Basin. Proceedings of the Geologists' Association, London, 100, 85-90.

Kethley, J.B., Norton, R.A., Bonamo, P.M. and Shear, W.A. 1989. A terrestrial alicorhagid mite (Acari: Acariformes) from the Devonian of New York. Micropaleontology, 35, 367-373.

Klimov, P., OConnor, B.M., Chetverikov, P.E., Bolton, S.J., Pepato, A.R., Mortazavi, A.L., Tolstikov, A.V., Bauchan, G.R. and Ochoa, R. 2018. Comprehensive phylogeny of acariform mites (Acariformes) provides insights on the origin of the four-legged mites (Eriophyoidea), a long branch. Molecular Phylogenetics and Evolution, 119, 105-117.

Lehtinen, P.T. 1991. Phylogeny and zoogeography of the Holothyrida. In: Dusbabek, F. and Bukva, V. (eds.), Modern Acarology. SPB Academic Publishing, Den Haag, 101-113.

Lourenço, W.R. 2001. A remarkable scorpion fossil from the amber of Lebanon. Implications for the phylogeny of Buthoidea. Comptes Rendus de l'Académie des Sciences, Paris, 332, 641-646.

Lourenço, W.R. 2002. The first fossil scorpion from the Cretaceous amber of Myanmar (Burma). New implications for the phylogeny of Buthoidea. Comptes Rendus Palevol, 1, 97-101.

Lourenço, W.R. 2016. A preliminary synopsis on amber scorpions with special reference to Burmite species: an extraordinary development of our knowledge in only 20 years. ZooKeys, 600, 75-87.

Lourenço, W.R. and Gall, J.-C. 2004. Fossil scorpions from the Buntsandstein (Early Triassic) of France. Comptes Rendus Palevol, 3, 369-378.

Lozano-Fernandez, J., Tanner, A.R., Vinther, J., Giacomelli, M., Carton, R., Edgecombe, G.D. and Pisani, D. 2019. Increasing species sampling in chelicerate genomic-scale datasets provides support for monophyly of Acari and Arachnida. Nature Communications.

Magalhaes, I.L.F., Azevedo, G.H.F., Michalek, P. and Ramirez, M.J. 2019. The fossil record of spiders revisited: implications for calibrating trees and evidence for a major faunal turnover since the Mesozoic. Biological Reviews, doi:10.1111/brv.1259.

Mans, B. J., de Klerk, D., Pienaar, R., Castro, M. H. and Latif, A. A. 2012. The mitochondrial genomes of *Nuttalliella namaqua* (Ixodoidea: Nuttalliellidae) and *Argas africolumbae* (Ixodoidea: Argasidae): estimation of divergence dates for the major tick lineages and reconstruction of ancestral blood-feeding characteristics. PLoS ONE 7(11), e4946.

Menon, F. 2007. Higher systematics of scorpions from the Crato Formation, Lower Cretaceous of Brazil. Palaeontology, 50, 185-195.

Michalik, P. and Ramírez, M.J. 2014. Evolutionary morphology of the male reproductive system, spermatozoa and seminal fluid of spiders (Araneae, Arachnida) — Current knowledge and future directions. Arthropod Structure & Development, 43, 291-322.

Norton, R.A., Bonamo, P.M., Grierson, J.D. and Shear, W.A. 1988. Oribatid mite fossils from a terrestrial deposit near Gilboa, New York. Journal of Paleontology, 62, 259-269.

Oliviera, I.S., Bai, M., Jahn, H., Gross, V., Martin, C., Hammel, J.U., Zhang, W. and Maer, G. 2016. Earliest onychophoran in amber reveals Gondwanan migration patterns. Current Biology, 26, 2594-2601.

Penney, D. Selden, P.A. 2002. The oldest linyphiid spider, in Lower Cretaceous Lebanese amber (Ananeae, Linyphiidae, Linyphiinae). Journal of Arachnology, 30, 487-493.

Pepato, A.R. and Klimov, P.B. 2015. Origin and higher-level diversification of acariform mites — evidence from nuclear ribosomal genes, extensive taxon sampling, and secondary structure alignment. BMC Evolutionary Biology (2015), 15, 178.

Perry, M.L. 1995. Preliminary description of a new fossil scorpion from the middle Eocene Green River Formation, Rio Blanco County, Colorado. In: Averettt, W.R. (ed.), The Green River Formation in Piceance Creek and Eastern Uinta Basins, 131-133. Grand Junction Geological Society, Grand Junction, CO.

Poinar, G. 2008. *Palaeosiro burmanicum* n. gen. n. sp., a fossil Cyphophthalmii (Arachnida: Opiliones: Sironidae) in Early Cretaceous Burmese amber. In: Makarov, S.E. and Dimitrijevićc (eds.), Advances in Arachnology and Developmental Biology. Papers dedicated to Prof. Dr. Božidar Ćurčić. Institute of Zoology, Belgrade, BAS. Sofia, Faculty of Life Sciences, Vienna, Monographs 12, 267-274.

Poinar, Jr., G. and Brown, A.E. 2003. A new genus of hard ticks in Cretaceous Burmese amber (Acari: Ixodida: Ixodidae). Systematic Parasitology, 54, 199-205.

Poinar, Jr., G. and Buckley, R. 2008. *Compluriscutula vetulum* (Acari: Ixodida: Ixodidae), a new genus and species of hard tick from Lower Cretaceous Burmese amber. Proceedings of the Entomological Society of Washington, 110, 445-450.

Raven, R.J. 1985. The spider Infraorder Mygalomorphae (Araneae): cladistics and systematics. Bulletin of the American Museum of Natural History, 390, 1-374.

Regier, J.C., Shultz, J.W., Zwick, A., Hussey, A., Ball, B., Wetzer, R., Martin, J.W., and Cunningham, C.W. 2010. Arthropod relationships revealed by phylogenomic analysis of nuclear protein-coding sequences. Nature, 463, 1079–1083.

Richardson, J.B., Bonamo, P.M. and McGregor, D.C. 1993. The spores of *Leclercqia* and the dispersed spore morphon *Acinosporites lindlarensis* Riegel: a case of gradualistic evolution. Bulletin of the Natural History Museum, Geology Series, 49, 121-155.

Santiago-Blay, J.A., Soleglad, M.E. and Fet, V. 2004. A redescription and family placement of *Uintascorpio* Perry, 1995 from the Parachute Creek Member of the Green River Formation (Middle Eocene) of Colorado, USA (Scorpiones: Buthidae). Revista Ibérica de Aracnología, 10, 7-16.

Schmidt, A.R., Jancke, S., Lindquist, E.E., Ragazzi, E., Roghi, G., Nascimbene, P.C., Schmidt, K., Wappler, T. and Grimaldi, D.A. 2012. Arthropods in amber from the Triassic Period. Proceedings of the National Academy of Sciences, USA, 109, 14796-14801.

Selden, P.A. 2002. First British Mesozoic spider, from Cretaceous amber of the Isle of Wight, southern England. Palaeontology, 45, 973-983.

Selden, P.A. 2012. A redescription of *Juraraneus rasnitsyni* Eskov, 1984 (Araneae: Juraraneidae), from the Jurassic of Russia. Bulletin of the British Arachnological Society, 15, 315-321.

Selden, P.A., Anderson, H.M. and Anderson, J.M. 2009. A review of the fossil spiders (Araneae) with special reference to Africa, and description of a new specimen from the Triassic Molteno Formation of South Africa. African Invertebrates, 50, 105-116.

Selden, P.A., Anderson, J.M., Anderson, H.M. and Fraser, N.C. 1999. Fossil araneomorph spiders from the Triassic of South Africa and Virginia. Journal of Arachnology, 27, 401-414.

Selden, P.A., Dunlop, J.A., Giribet, G., Zhang, W. and Ren, D. 2016. The oldest armoured harvestman (Arachnida: Opiliones: Laniatores), from the Upper Cretaceous Myanmar amber. Cretaceous Research, 65, 206-212.

Selden, P.A. and Gall, J.-C. 1992. A Triassic mygalomorph spider from the northern Vosges, France. Palaeontology, 35, 211-235.

Selden, P.A. and Huang, D. 2010.The oldest haplogyne spider (Araneae: Plectruridae) from the Middle Jurassic of China. Naturwissenschaften, 97, 449-459.

Selden, P.A. and Penney, D. 2010. Fossil spiders. Biological Reviews, 85, 171-206.

Selden, P.A., Shih, C. and Ren, D. 2011. A golden orb-weaver spider (Araneae: Nephilidae: Nephlia) from the Middle Jurassic of China. Biology Letters, 7, 775-778.

Selden, P.A., Shih, C. and Ren, D. 2013. A giant spider from the Jurassic of China reveals greater diversity of the orbicularian stem group. Naturwissenschaften, 100, 1171-1181.

Sharma, P.P., Baker, C.M., Cosgrove, J.G., Johnson, J.E., Oberski, J.T., Raven, R.J., Harvey, M.S., Boyer, S.L. and Giribet, G. 2018. A revised dated phylogeny of scorpions: Phylogenomic support for ancient divergence of the temperate Gondwanan family Bothriuridae. Molecular Phylogenetics and Evolution, 122, 37-45.

Sharma, P.P., Fernández, R., Esposito, L.A., González-Santillán, E. and Monod, L. 2015. Phylogenomic resolution of scorpions reveals multilevel discordance with morphological phylogenetic signal. Proceedings of the Royal Society, B, 282, 20142953.

Sharma, P.P. and Giribet, G. 2014. A revised dated phylogeny of the arachnid order Opiliones. Frontiers in Genetics, 5, 255, 1-13.

Shi, G., Grimaldi, D.A., Harlow, G.E., Wang, J., Wang, J., Yang, M., Lei, W., Li, Q., and Li, X. 2012. Age constraint on Burmese amber based on U–Pb dating of zircons. Cretaceous Research, 37, 155-163.

Shultz, J.W. 1998. Phylogeny of Opiliones (Arachnida): An assessment of the "Cyphopalpatores" concept. Journal of Arachnology, 26, 257-272.

Shultz, J.W. 2007. A phylogenetic analysis of the arachnid orders based on morphological characters. Zoological Journal of the Linnean Society, 150,221-265.

Sidorchuk, E.A., Schmidt, A.R., Ragazzi, E., Roghi, G. and Lindquist, E.E. 2014. Plant-feeding mite diversity in Triassic amber (Acari: Tetrapodili). Journal of Systematic Palaeontology, 13, 129-151.

Smith, M.E., Singer, B. and Carroll, A. 2003. $^{40}Ar/^{39}Ar$ geochronology of the Eocene Green River Formation, Wyoming. Geological Society of America Bulletin, 115, 549-565.

Soleglad, M.E., Fet, V., and Kovařik, F. 2005. The systematic position of the scorpion genera *Heteroscorpion* Birula, 1903 and *Urodacus* Peters, 1861 (Scorpiones: Scorpionoidea). Euscorpius, 2005, 1-37.

Subillas, L.S. and Arillo, A. 2002. Oribatid mite fossils from the Upper Devonian of South Mountain, New York and the Lower Carboniferous of County Antrim, Northern Ireland (Acariformes, Oribatida). Estudios del Museo de Ciencias Naturales de Álava, 17, 93-106.

Wellman, C.H. and Richardson, J.B. 1993. Terrestrial plant microfossils from Silurian inliers of the Midland Valley of Scotland. Palaeontology, 36, 155-193.

Wheeler, W.C., Coddington, J.A., Crowley, L.M., Dimitrov, D., Goloboff, P.A., Griswold, C.E., Hormiga, G., Prendini, L., Ramírez, M.J., Sierwald, P., Almeida-Silva, L., Alvarez-Padilla, F., Arnedo, M.A., Benavides Silva, L.R., Benjamin, S.P., Bond, J.E., Grismado, C.J., Hasan, E., Hedin, M., Izquierdo, M.A., Labarque, F.M., Ledford, J., Lopardo, L., Maddison, W.P., Miller, J.A., Piacentini, L.N., Platnick, N.I., Polotow, D., Silva-Dávila, D., Scharff, N., Szuts, T., Ubick, D., Vink, C,J., Woodm H.M. and Zhang, J. 2017. The spider tree of life: phylogeny of Araneae based on target-gene analyses from an extensive taxon sampling. Cladistics, 33, 574-616.

Wilson, H.M. 2005. Zosterogrammida, a new order of millipedes from the middle Silurian of Scotland the Upper Carboniferous of Euramerica. Palaeontology, 48, 1101-1110.

Wolfe, J.M., Daley, A.C., Legg, D.A. and Edgecombe, G.D. 2016. Fossil calibrations for the arthropod Tree of Life. Earth-Science Reviews, 160, 43-110.

Zwick, A., Regier, J.C. and Zwickl, D.J. 2012. Resolving Discrepancy between Nucleotides and Amino Acids in Deep-Level Arthropod Phylogenomics: Differentiating Serine Codons in 21-Amino-Acid Models. PLoS ONE, 7, e47450.
